# Enhancing the Implementation and Integration of mHealth Interventions in Resource-Limited Settings: A Scoping Review

**DOI:** 10.21203/rs.3.rs-4757157/v1

**Published:** 2024-07-19

**Authors:** Wilson Tumuhimbise, Stefanie Theuring, Fred Kaggwa, Esther C Atukunda, John Rubaihayo, Daniel Atwine, Juliet N Sekandi, Angella Musiimenta

**Affiliations:** Faculty of Computing and Informatics, Mbarara University of Science and Technology, Mbarara Uganda; Institute of International Health, Charité-Universitätsmedizin Berlin, corporate member of Freie Universität Berlin and Humboldt-Universität zu Berlin, Germany; Faculty of Computing and Informatics, Mbarara University of Science and Technology, Mbarara Uganda; Faculty of Medicine, Mbarara University of Science and Technology, Mbarara Uganda; Faculty of Health Sciences, Mountains of the Moon University, Fort Portal, Uganda; Soar Research Foundation, Mbarara, Uganda; Global Health Institute, University of Georgia, Georgia, USA; Faculty of Computing and Informatics, Mbarara University of Science and Technology, Mbarara Uganda, Angels Compassion Research and Development Initiative, Mbarara Uganda

**Keywords:** mHealth, implementation, integration, resource-limited settings, framework

## Abstract

**Background.:**

Although mobile health (mHealth) interventions have shown promise in improving health outcomes, most of them rarely translate to scale. Prevailing mHealth studies are largely small-sized, short-term and donor-funded pilot studies with limited evidence on their effectiveness. To facilitate scale-up, several frameworks have been proposed to enhance the generic implementation of health interventions. However, there is a lack of a specific focus on the implementation and integration of mHealth interventions in routine care in low-resource settings. Our scoping review aimed to synthesize and develop a framework that could guide the implementation and integration of mHealth interventions.

**Methods:**

We searched the PubMed, Google Scholar, and ScienceDirect databases for published theories, models, and frameworks related to the implementation and integration of clinical interventions from 1^st^ January 2000 to 31^st^ December 2023. The data processing was guided by a scoping review methodology proposed by Arksey and O’Malley. Studies were included if they were i) peer-reviewed and published between 2000 and 2023, ii) explicitly described a framework for clinical intervention implementation and integration, or iii) available in full text and published in English. We integrated different domains and constructs from the reviewed frameworks to develop a new framework for implementing and integrating mHealth interventions.

**Results:**

We identified eight eligible papers with eight frameworks composed of 102 implementation domains. None of the identified frameworks were specific to the integration of mHealth interventions in low-resource settings. Two constructs (skill impartation and intervention awareness) related to the training domain, four constructs (technical and logistical support, identifying committed staff, supervision, and redesigning) from the restructuring domain, two constructs (monetary incentives and nonmonetary incentives) from the incentivize domain, two constructs (organizational mandates and government mandates) from the mandate domain and two constructs (collaboration and routine workflows) from the integrate domain. Therefore, a new framework that outlines five main domains—train, restructure, incentivize, mandate, and integrate (TRIMI)—in relation to the integration and implementation of mHealth interventions in low-resource settings emerged.

**Conclusion:**

The TRIMI framework presents a realistic and realizable solution for the implementation and integration deficits of mHealth interventions in low-resource settings.

## Background

Mobile health (mHealth) is defined as a healthcare delivery system carried out via mobile devices to enable better healthcare access and to support the performance of health workers [1]. It facilitates remote access to previously hard-to-access specialized healthcare services [2]. Mobile apps are among the most often used mHealth interventions [3, 4] and have the potential to provide users with affordable access to high-quality and evidence-based health information [5]. The number of mobile phone subscribers in low- and middle-income countries (LMICs) has continued to increase, with over 6.91 billion users as of 2021 [6], surpassing the total population of 6.61 billion people in LMICs [7]. This exponential growth in subscription offers an opportunity for the utilization of mobile applications (apps), short messaging service (SMS) reminders, or wearable devices (smartwatches, armbands) in mobile-based interventions in healthcare.

Implementation is defined as the “social organization of bringing a practice or practices into action”, while integration is defined as “the process by which a practice or practices are reproduced and sustained among the social matrices of an organization or institution” [8]. Loman and colleagues defined sustainability as the “continued implementation of a practice at a level of fidelity that continues to produce intended benefits” [9]. For interventions to be considered successful, efforts to ensure continued use as planned to achieve the intended benefits are crucial for implementers to consider. Otherwise, the lack thereof causes these interventions to stagnate.

The adoption of mHealth interventions in routine care remains minimal, especially in low-resource settings, where the majority of these interventions have not been scaled up as expected [10]. This limited scale-up can be attributed to small short-term pilot studies funded by donors, limited understanding between mHealth and key stakeholders, taxation, or a perceived lack of evidence among donors and governments about the effectiveness of mHealth [11]. Additionally, mHealth intervention utilization is still limited by evolving technologies due to the frequent release of new devices and platforms [12] and incompatibility issues that affect proper functionality [13]. Several other factors, such as upgrades on these platforms, are beyond the developer’s control and affect the stability of these interventions. On the other hand, the selection of mobile phones on which these interventions run raises several questions of whether to provide users with phones to use the intervention or to install an intervention on the user’s phones; the former may be costly in terms of procuring new mobile devices, and the latter might face incompatibility issues and might decrease the frequency of usability [13]. All these issues present unique requirements for the utilization of mHealth interventions compared to other interventions.

The complex nature of healthcare systems, characterized by busy schedules while dealing with patients [14], lack of motivation [15] and fatigue [16] in low-resource settings, may bury life-changing mHealth interventions that could otherwise improve healthcare outcomes. Healthcare systems, especially in LMICs, are characterized by disorganized leadership structures [17], a high doctor-to-patient ratio (1.3 per 1000 compared to the WHO-recommended 2.5 per 1000 [18]) and an already overburdened health sector [19]. Moreover, additional requirements for the use of mHealth interventions can potentially increase technological fatigue and extra workload for healthcare workers. The development of interventions that do not address the factors highlighted above may render mHealth interventions useless due to a lack of uptake and implementation.

The healthcare landscape is constantly evolving and driven by organizational needs and national priorities. As innovative technologies, such as mHealth interventions, become increasingly common, their usability seems almost inevitable. However, integrating these mHealth interventions into routine healthcare has proven challenging due to lack of frameworks specifically designed to guide this process [20]. Successful implementation hinges on addressing these integration barriers. Therefore, without a well-defined process for their implementation and integration, these mHealth interventions risk failing to deliver their intended benefits. The existing literature offers a wealth of generic theories and frameworks [21–24] to guide intervention implementation. Although valuable, these frameworks lack the specificity required for mHealth interventions. This gap highlights the need for a coherent framework specifically tailored to the sustainable implementation and integration of mHealth interventions in resource-constrained settings. Locally contextualized frameworks that target existing barriers have the potential to significantly improve the success rate of well-intentioned mHealth interventions. Without a clear and well-defined implementation and integration plan, these interventions are more likely to fail, resulting in wasted financial resources for implementers, funders, and governments.

This investigation does not replace these frameworks but aims to integrate domains and constructs from these existing frameworks to present a specific framework that can guide the implementation and integration of mHealth interventions in low-resource settings. This research therefore seeks to i) review the existing frameworks/models/theories for intervention implementation to understand the state of the art regarding the implementation and integration aspects of mHealth interventions and ii) formulate a framework for guiding the implementation and integration of mHealth interventions based on the identified domains.

## Methodology

The Arksey and O’Malley scoping review methodology was used to include diverse study types [25]. The methodology outlines six main steps that should be followed: i) identifying the research question, ii) identifying relevant studies, iii) selecting studies, iv) charting data, v) collating, summarizing and reporting results, and vi) consulting. The sixth step, which involved consulting stakeholders, was not considered for this review. We followed a purposive search strategy for identifying, screening and analyzing relevant studies that discussed frameworks, models or theories for implementing and integrating mHealth interventions. This review was reported in accordance with the Preferred Reporting Items for Systematic Reviews and Meta-Analyses (PRISMA) extension for scoping reviews [26] checklist for guiding the presentation of the findings. There is no published protocol for this review. This review involved two main steps: i) examining the literature to identify existing frameworks and ii) developing a new framework to guide the implementation and integration of mHealth interventions.

### Identification of the Research Question

This study was guided by two main research questions: *i) what are the existing frameworks, models or theories for implementing and integrating clinical interventions, and ii) how can different domains/constructs of these frameworks be integrated to formulate a new framework for implementing and integrating mHealth interventions?*

### Identification of Relevant Studies

To develop a specific framework for implementing and integrating mHealth interventions in routine clinical settings, we conducted a comprehensive literature review. Our goal was to identify and understand existing frameworks, models, and theories related to the implementation of clinical interventions in general, with a particular focus on behavioral aspects. The search strategy aimed to capture the current state of the art by identifying articles that discussed implementation and integration frameworks for clinical interventions, specifically those mentioning mHealth interventions. We conducted the search in December 2023 using the Google Scholar, PubMed, and ScienceDirect databases. Our search terms included combinations of “framework,” “model,” or “theory” with “implementation” or “integration,” alongside “intervention” and “mHealth.” To ensure a thorough review, we also searched the reference lists of identified articles for additional relevant studies. EndNote X7 (Thomson Reuters, Philadelphia, PA, USA) was used to manage and organize the search results.

### Study Selection

Studies were included if they were i) peer reviewed, ii) explicitly described a framework for clinical intervention implementation and integration—we defined implementation as the process of putting to use or integrating interventions [27], iii) implemented and integrated strategies, iv) published between 2000 and 2023, or v) available and published in the English language. Studies were excluded if they did not report the development of a framework for the implementation or integration of clinical interventions or were carried out before 2000. All the studies were explicitly scrutinized to ensure that they reported implementation or integration frameworks for clinical intervention; therefore, we did not include protocols or formative/exploratory studies. We excluded studies that solely applied existing frameworks unless they presented a novel framework within the discussed intervention [28].

### Charting of the Data

Reviewers WT and AM assessed the titles and abstracts to identify relevant articles that met the inclusion criteria. In instances where the reviewers were not sure about the articles, members of the team were invited to discuss them at length and to conclude. The following characteristics were extracted from the included studies: the aim of the framework, different domains and constructs addressed by the framework and the definition of the domains. A table (Table 1) was used to incorporate all the extracted data.

### Collating, summarizing, and reporting results

The research team had a series of iterative discussions and reviews to analyze and agree on the final articles to be included in this scoping review. The key characteristics are tabulated in Table 1 to capture the most important details of the identified frameworks.

### Development of the mHealth intervention implementation and integration framework

Guided by the constructionist paradigm that asserts that realities are a social construction of one’s own mind [29]. This development of the new framework culminated from the implementation lessons learnt from the first author’s (WT) PhD research [30–33] that sought to implement a mobile health application for following up presumptive TB patients referred from private to public hospitals in Uganda. During the implementation process, a few challenges like lack of use due to busy schedules and lack of internet that hindered usability were noted. These are challenges that cut across during the implementation of well-intended mHealth interventions. This triggered our quest to develop a framework that can guide on what needs to be done as far as implementing and integrating mHealth interventions in low resource settings is concerned. To make evidence based conclusions, a review of published implementation frameworks was carried out for potential domains, constructs and explanation for rationalization.

Our new framework draws upon key domains and constructs identified within existing frameworks that demonstrably facilitate mHealth implementation and integration. We employed a content analysis approach to systematically extract these crucial components. This iterative process involved ongoing discussion and review by all the authors until the final set of domains and constructs was established. The resulting framework integrates valuable insights gleaned from previously reviewed frameworks. It emphasizes the critical multilevel factors that must be addressed to ensure successful, sustainable implementation and integration of mHealth interventions.

### Author Reflexivity Statement

This work culminated from the implementation lessons learnt from the first author’s (WT) PhD research [30–33] that sought to implement a mobile health application for following up presumptive TB patients referred from private to public hospitals in Uganda. This work was supervised by authors AM (Senior Lecturer and mHealth Implementation researcher), FK (Senior Lecture and Computer Scientist) and, DA (Senior Implementation Research and Epidemiologist) at Mbarara University of Science and Technology, Uganda. During this research, a mobile application called Tuuka app [31] was pilot tested among 22 healthcare workers for following up presumptive TB patients referred from private to public hospitals in southwestern Uganda, however during the implementation process, a few challenges were noted for example, lack of use due to busy schedules and lack of internet that hindered usability. These are challenges that cut across during the implementation of well-intended mHealth interventions. This triggered WT’s quest whose background is health informatics and mHealth implementation to define a potential framework that can guide implementers on what should be done as far as implementing and integrating mHealth interventions in low resource settings is concerned, given its unique needs. The development of this framework was informed by a constructivist paradigm that asserts that realities are a social construction of one’s own mind [29]. This was supplemented by a thorough review of the published implementation frameworks for identification and explanation of domains related to our own framework for rationalization. Therefore the researchers already had a few preconceived constructs that they wanted to present in an evidence-based structured manner and were aware of the technical terminology required to inform implementers and policy makers regarding mHealth implementation and integration in low resource settings. Although this was our best methodological approach to the best of our knowledge in formulation of this framework, there could have been approaches that would have reinforced the framework. The researchers are aware of the risks associated with this approach for example imposing the researcher’s own beliefs and perceptions in developing an implementation framework. However, authors ST (Senior Research Associate at the Institute of International Health, Charité-Universitätsmedizin Berlin), JNS (Associate Professor at the Global Health Institute, University of Georgia, Georgia, USA), ECA (Senior Lecturer and mHealth Implementation Researcher at the Faculty of Medicine at MUST) and JR (Professor of Public Health at the Faculty of Health Sciences, Mountains of the Moon University, Fort Portal, Uganda) provided valuable feedback that helped refine the framework’s domains, constructs, and overall guidance for mHealth implementation and integration in low-resource settings.

## Results

**Figure 1:**
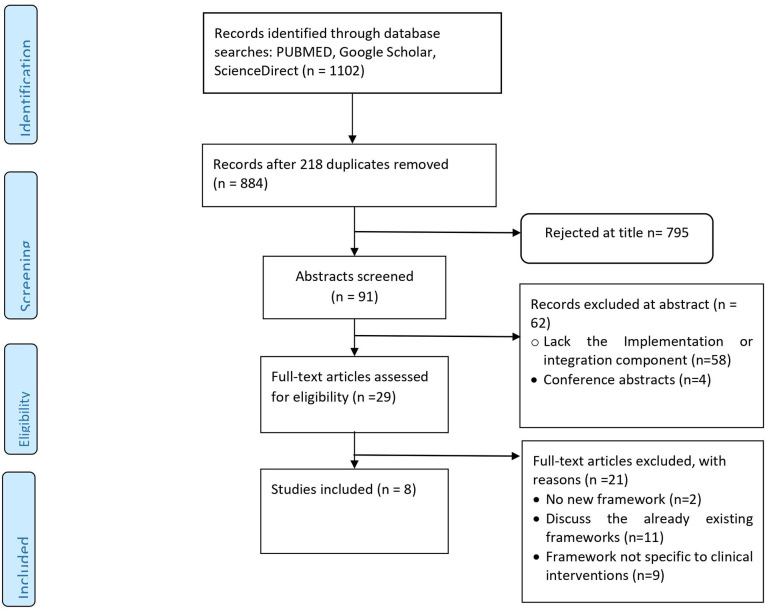
Flow diagram for the selected studies

The database search identified 1102 articles, of which 218 duplicates were removed (Fig. 1). A total of 795 articles were eliminated after title screening, and an additional 62 articles were excluded after full abstract screening. Twenty-one articles were excluded upon examination of the full texts. Therefore, eight studies were ultimately included in the analysis, as shown in Table 1 below.

We identified seven main frameworks, namely, the Consolidated Framework for Implementation Research (CFIR) [22, 34], the modified Consolidated Framework for Implementation Research (mCFIR) [35], the Unified Theory of Acceptance and Use of Technology (UTAUT) model [21], the Capability Opportunity and Motivation and Behavior (COM-B) Model [23], the Reach, Efficacy, Adoption, Implementation, and Maintenance (RE-AIM) framework [36], the Normalization Process Theory (NPT) [8], and the theory of organizational readiness for change [37]. In addition to the reviewed frameworks, the Expert Recommendations for Implementing Change (ERIC) proposed by Powell and colleagues [38], though not characterized as a framework, was also considered during the development of the framework. The reviewed frameworks synthesize several other published studies and frameworks. For example, CFIR synthesizes 19 implementation theories, the UTAUT model is a unification of eight technology acceptance models, and the COM-B model synthesizes 19 behavioral theories, which strengthens the tailoring of the TRIMI framework. Table 1 below provides an overview of the frameworks reviewed and their domains.

Out of the eight frameworks reviewed, only three comprehensively described training as important component and explicitly provided descriptions in relation to mHealth implementation and integration and these are; i) the COM-B model [23] that defines training and education as intervention functions components of the capability and motivation domains of the model for developing/imparting skills and imparting knowledge to use the intervention; ii) the ERIC [38] that underscores conducting ongoing training, carrying out educational meetings targeted towards different stakeholders to teach them about the intervention as potential implementation strategies; and iii) the mCFIR framework [35] that highlights the role of training health care practitioners in using digital health interventions.

Five reviewed frameworks comprehensively defined restructuring to enable the implementation and integration of mHealth interventions and these are; i) the COM-B model[23] that defines environmental restructuring an intervention function for both the capability and motivation domains as changing the physical or social context for the intervention to be implemented successfully; ii) the ERIC [38] that highlights centralizing and providing local technical assistance, changing the liability laws, changing the physical structure and equipment, using data experts, and providing clinical supervisions as key implementation strategies for mHealth implementation and integration; iii) the UTAUT model [21] that defines the facilitating conditions that is defined as the degree to which an individual believes that an organizational and technical infrastructure exists to support the use of the system; iv) the REAIM [24] that defines maintenance domain as the extent to which a program is sustained over time; and v) the theory of organizational readiness for change [37] that highlights change valence which is the degree to which members of the organization value the forthcoming change and contextual factors that refers to the degree to which an organization’s culture embraces an intervention which are the key tenets of restructuring.

Two reviewed frameworks explicitly defined the components of incentivisation as a key factor for implementing and integrating mHealth and these are: i) COM-B model [23] that underscores incentivisation an intervention function of the motivation domain as creating expectation for reward; ERIC [38] that highlights incentivizing the adoption of and implementation of the intervention, the development of disincentives that involves provision of financial disincentives upon failure to use the intervention.

Only framework underscored the role of mandating change that involves having leadership declare the priority of the innovation and their determination to have it implemented [38]. On the other hand, the integration component of implementing and integrating mHealth interventions was comprehensively discussed by three reviewed frameworks and these are: i) the CFIR framework [22, 34] that highlights the compatibility and absorptive capacity constructs in the inner domain about how well the intervention aligns with the organization’s ability to absorb and integrate the new intervention, additionally, CFIR highlights the planning construct that describes the extent to which an organization plans and prepares tasks for implementing an intervention are developed in advance. ii) the NPT framework that describes the cognitive participation domain that involves engaging human actors to use the intervention which is key in utilizing the intervention.

## Overview of the TRIMI domains and constructs

**Figure 2:**
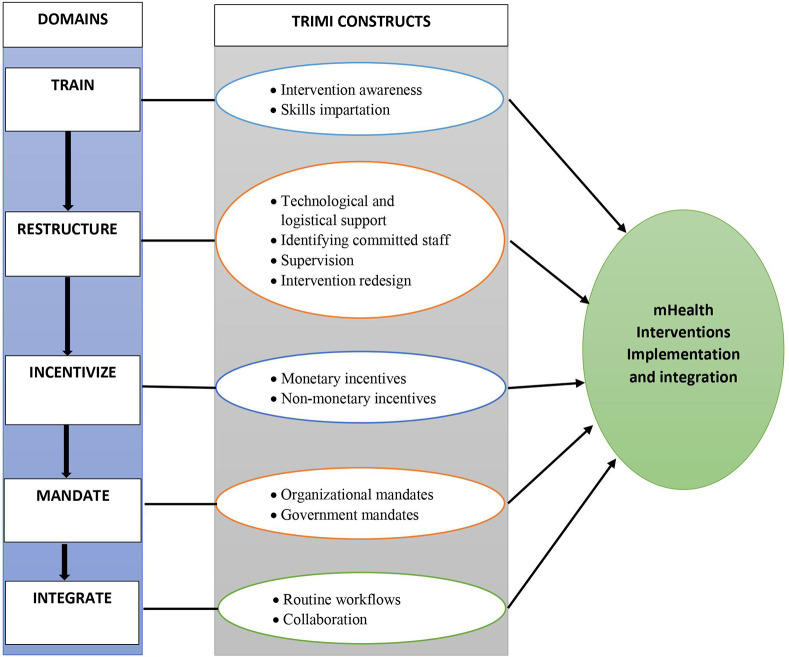
Overview of the TRIMI Domains and constructs

For our developed framework (Fig. 2), we integrate different domains and constructs from the above frameworks in Table 1, which focus on guiding the implementation and integration of mHealth interventions. The training domain emerged from the COM-B model, ERIC, and mCFIR; the restructuring domain emerged from the COM-B model, ERIC, UTAUT, RE-AIM and theory of organizational readiness for change; the incentivization domain emerged from the COM-B model and ERIC; the mandate domain emerged from ERIC; and the integration domain emerged from the NPT and CFIR, as shown in Table 1 above. Therefore, the TRIMI framework is composed of five key domains through which the successful implementation and integration of mHealth innovations can be affected, namely, train, restructure, incentivize, mandate, and integrate as shown in Table 2 below.

### Domain 1: Train

This domain is aimed at empowering and educating users about the importance of utilizing the intervention. This is key to increasing awareness about mHealth interventions to ensure their adequate use [39] and equipping clinical supervisors who will supervise other users with technical skills to use the intervention [38]. We categorized this domain into two main constructs:

- Intervention awareness is the degree to which users become aware of an intervention [40]. Awareness is key in enhancing intervention diffusion. Developers and implementers can organize workshops and seminars aimed at making system users and key stakeholders aware of mHealth interventions and their importance.
- Skills impartation aimed at equipping the intervention users with technical knowledge and skills [23] for using the mHealth intervention. It should be noted that being aware of an intervention is not enough if users lack the skills to effectively use the intervention. Individual or group sessions with users by trained professionals to instill confidence in utilizing the intervention can enhance the impartation of skills among users [41]. This training should be aimed at empowering key intervention users about the details of the intervention and showing them how the intervention works. User manuals highlighting the importance and use of the intervention can be developed to enable intervention users to become acquainted with the system.

This domain suggests that implementers should expend careful effort in establishing appropriate awareness and training mechanisms that will engage users in the intervention. During these sessions, information should be presented using mechanisms that enable recalling and retention [39], which can be a combination of metaphors and mindfulness approaches with a series of practical, hands-on exercises [42].

### Domain 2: Restructure

This domain is aimed at improving or changing the physical or social context around an individual or a healthcare facility to influence their use of the intervention. We categorized this domain into four main constructs

- Technical and logistical support involves the provision of technical help for users to ensure continued functionality of the intervention [43]. The level of support offered to users determines the quality of user interaction. Centralizing technical assistance aimed at dealing with technical issues, such as application reinstallations due to accidental deletions [44], that may arise is a key implementation strategy for clinical interventions [38]. Logistical support, on the other hand, involves the provision of logistics such as dedicated internet services, alternative charging systems, and smartphones to intervention users to facilitate ease of use of the mHealth intervention. Venkatesh and colleagues noted the role of facilitating conditions in enhancing technology acceptance and usage [21].
- Identifying committed staff, which can be made possible by hiring new staff dedicated to the operationalization of the intervention if necessary, can also act as change agents to support the intervention implementation. These individuals can play a role in preparing health facilities for intervention implementation and integration by garnering commitment from various stakeholders, including the government, private sector, and other funding bodies, to ensure continued funding for system implementation [45]. Limited commitment from key stakeholders hinders efforts to implement interventions within health facilities; thus, there is a need for unwavering and persistent commitment to have these key stakeholders brought on board [37].
- Supervision involves monitoring users on a routine basis specifically for addressing any issues regarding the use of the intervention. It also involves helping users handle any tricky situation that could emerge during the implementation process [46]. Supervision ensures correct usage of the intervention in the case of nontechnical public healthcare practitioners; therefore, providing users with routine supervision regarding the developed intervention plays a role in successful implementation and integration [38].
- Intervention redesign is a key component for enhancing and managing the recurring intervention design issues that may arise from users and enable accommodation of future changes. These include fixing bugs, system upgrades, changing the layout, and ensuring compatibility with emerging technologies and platforms such as operating systems and mobile devices.

This domain therefore suggests that organizational restructuring involving technical and logistical support, identifying committed staff, supervision and intervention redesign could enhance the implementation and integration of mHealth interventions.

### Domain 3: Incentivize

This domain is aimed at motivating users to use the intervention. Incentivization is defined as the practice of creating and serving an expectation for reward and has been proven to influence the behavior of using a given intervention [23]. Incentivization can be financial (monetary) or nonfinancial (nonmonetary) depending on the project’s design. We therefore categorize this domain into two main constructs

- Monetary incentivization involves the provision of direct conditional or unconditional financial incentives to intervention users upon reaching a certain milestone of using an intervention. Implementers should devise means of tagging monetary incentives with interventions, for example, a mobile money-based intervention to support access and adherence to tuberculosis medication in southwestern Uganda, where tuberculosis patients receive transport refunds, and a monthly adherence incentive upon attaining a percentage adherence greater than or equal to 90% was perceived to be useful in proving their commitment to healthcare workers [47].
- Nonmonetary incentivization is the provision of nonfinancial incentives for acknowledging best intervention users and promoting team-based performance. These incentives are aimed at rewarding and appreciating high-level achievement and performance after a predetermined goal is achieved [48]. This is key in motivating the use of an intervention in an instance where monetary incentivization is not possible. A carrot reward application aimed at rewarding users with loyalty points for downloading it, referring friends to download the app, and completing a quiz resulted in higher engagement levels in Canada [49].

This domain underscores the need to ensure that users are motivated to continuously utilize the intervention. Powell and colleagues highlighted the need to continuously alter incentives or allowance structures to motivate those who use the intervention well instead of deducting it from those who do not utilize it well. Therefore, financial disincentivization involves removing financial incentives from users for failing to use the intervention as a potential remedy to motivate continued use [38]. Therefore, this domain suggests that the incorporation of both monetary and nonmonetary incentives could enhance the usability of the intervention among users.

### Domain 4: Mandate

This domain is aimed at mandatory authorization to use the intervention. It is meant to address issues related to resistance to change and lack of trust among users. We categorized this domain into two constructs:

- Organizational mandates that involve internal policies and procedures within a healthcare facility aimed at ensuring that the implementation of the intervention is carried out as intended. This requires buy-in from top management and other key relevant stakeholders in a healthcare facility. Implementers need to develop internal policies and procedures regarding the mandatory use of the intervention, including highlighting the benefits of using the intervention. Organizational policies play a role in enhancing technological awareness [50] and highlighting the need for urgency to implement the intervention and to prevent individuals from blocking the intervention [51].
- The government mandates, which we define as the extent to which the government or state agencies make compulsory use of a given intervention. It involves putting in place policies and procedures for ensuring that individuals, organizations, and facilities are utilizing the intervention as expected. In instances where the intervention is for the public good, the government may, through the respective ministries, departments, and agencies, implement guidelines aimed at compulsory utilization of the developed intervention.

It should be noted that some technologies that require compulsory usability have been perceived to violate human liberties and rights [52]. However, in terms of public health emergencies, delays in implementing readily available interventions may cause health and economic costs that would have been avoided. In instances of public health emergencies, the achievement of public health goals should take precedence, which may necessitate the applicability of the coercive powers of the state [53]. Therefore, despite the moral questions that may arise, several justificatory conditions (effectiveness, proportionality, necessity, least infringement, and public justification) have been proposed by [53] to determine whether the implementation of health interventions can override existing ethical considerations.

Although the mandatory installation of apps during public health emergencies is justifiable, there is a need for efforts to ensure that confidence is instilled among users to be able to use the application [52]. Notably, mandating only applies to instances where the intervention is of national or organizational or public importance, for example, an intervention supporting adherence to medication for an infectious disease given that nonadherence can result in spreading the disease to other people. Therefore, while mandates can be useful, they should be used judiciously and only for interventions with significant public benefit. User training can help address user concerns.

### Domain 5: Integrate

This domain suggests that the sustainable usability of the intervention among healthcare workers can be improved by integrating the intervention into routine workflows and collaborating with key stakeholders for buy-in about the implemented solution. We categorized this domain into two constructs

- Routine workflow integration is the degree to which the new technology can be embedded into the existing healthcare practice within a healthcare facility. This is key for intervention adoption. There is a need to ensure a careful study of how routines within a healthcare facility are carried out and how best an intervention can automate the work processes to enhance usability. For example, if a healthcare facility has been using paper-based data management, a new electronic intervention can be adopted for data management. The lack of meaningful integration with clinical systems has contributed to the failure of several mHealth initiatives [54]; thus, these interventions need to be carefully integrated into existing healthcare practices [55]. Damschroder and colleagues also highlight integration into work processes [34] as a key factor for intervention implementation. If an mHealth intervention is not yet integrated into routine care, it remains a platform instead of a solution for improving healthcare outcomes [56]. Therefore, this integration should be user-centered by ensuring that the intervention fits into existing workflows without creating an additional burden for healthcare workers.
- Collaboration refers to the degree to which relevant stakeholders (clinicians, patients, hospital administrators, funders) are brought on board to support mHealth intervention implementation. The integration of mHealth interventions can be enhanced by collaborative efforts from all relevant stakeholders within the mHealth ecosystem to help overcome barriers hindering healthcare delivery and disease management, thus garnering buy-in from all stakeholders [10].

## Discussion

We present the TRIMI framework, which can be used to guide the implementation and integration of mHealth interventions in healthcare facilities. The TRIMI framework proposes that for the sustainable implementation and integration of mHealth interventions to occur, intervention users must be trained, the usability environment should be restructured, users should be incentivized and mandated to use the intervention, and the intervention should be integrated within the routine workflow. Failure to meet these criteria may compromise the successful implementation and integration of the intervention. The framework was not developed in isolation but rather based on the already existing well-known implementation frameworks to derive guidance and build a trusted framework for sustainable implementation and integration of mHealth interventions, thus complementing the generic existing implementation frameworks and theories.

The TRIMI can be used for formative assessment before the implementation of the mHealth intervention to ascertain the degree to which the intervention will be implemented and integrated as desired. It can also be used to assess the motivators for mHealth implementation and integration if the factors are present within a healthcare facility and for barriers if they are lacking during implementation. However, it is important to monitor several contextual factors that may arise during the implementation of these domains [22] that might affect the usability, scalability, and sustainability of the intervention [57]; this necessitates the implementers to understand and devise ways in which factors can be addressed to lessen any negative impact they might have on the intervention implementation. Although we agree that mHealth is broad with different facets, including wearable technologies, SMS reminders, mobile applications, and phone calls, we believe that the TRIMI framework cuts across these categories to provide generic guidance for the sustainable implementation and integration of these interventions.

The role of training in the adoption of mHealth tools among clinicians has been clearly documented [58] due to its ability to impart skills and increase awareness about the intervention [23]. Training should be aimed at helping users of the intervention perform and fulfill specific tasks [59]. Interactive training accompanied by practical aspects regarding an intervention’s use has been reported as a key factor for its successful use. In their scoping review, Brunner and colleagues noted that training should focus on training users on how to use the intervention and support them for continuous use. This should be carried out in an interactive fashion that offers choices to users [60]. There is a need to develop training resources that are simple and easy to follow for supporting learning regarding the use of interventions that can be delivered in person or via the web in the form of videos and text-based resources [60]. The development of resources should follow a codesign approach with key users, including clinicians, hospital administrators, patients and all relevant stakeholders [61]. Therefore, the TRIMI proposes that training is a key pillar of the sustainable implementation of mHealth interventions. If the training needs of the key users are carefully taken into consideration, then their awareness regarding the intervention will increase, which will boost their desire to gain skills that will help them utilize the intervention.

The implementation of mHealth interventions requires careful reconsideration of the environment in which they are going to be utilized. It is therefore important to ensure that both the social and physical contexts are organized differently to facilitate the use of mHealth interventions. This not only prepares health facilities to adequately use the intervention but also allows individuals to act as change agents. However, restructuring is not without cost since it might involve hiring newly committed staff, reimbursing teams to carry out technological support, and redesigning the intervention in case of future user needs. This implies that for meaningful and intentional intervention implementation and integration to occur, financial costs may be inevitable. Therefore, health facilities may require additional finances to facilitate these aspects, which may be challenging in low-resource settings [62]. However, a cost‒benefit analysis can be carried out to weigh these options, especially if the benefits outweigh the risks but if the costs exceed the benefits, it is advised not to be undertaken [63]. Additionally, the financial incentives for facilitating the use of mHealth interventions as part of the incetivize domain may also not be a sustainable approach since they add a cost burden on the side of the health facility. Therefore, nonfinancial incentives offer a long-term sustainable approach for continued use of the intervention. In instances where funding is available, a mixture of both monetary and nonmonetary incentives has been shown to have a greater positive impact on performance [64].

The mandating domain, as suggested by the TRIMI framework in this regard, is not as negative as it may sound. It is the process of requiring a health facility to use an mHealth intervention to deliver healthcare services to patients and to support clinicians’ performance. A lack of legislation has been reported among the main reasons why healthcare workers do not use mobile applications [65]. Powell and colleagues also mention mandating change, which involves organizations declaring their prioritizing of the developed intervention and determination to have it implemented as one of the implementation strategies [38]. Therefore, ensuring the establishment of both organizational regulations regarding the use of mHealth interventions and compulsory government mandates on the adoption of the intervention may enhance usability and adoption. In the USA, the mandatory use of prescription drug monitoring programs (PDMPs), which store controlled substance dispensing information digitally and make it accessible to prescribers, pharmacies and law enforcement officers, has resulted in increased access to databases, a reduction in unsafe opioid prescriptions among early adopter states [66], and reduced rates of opioid use in patients [67]. The mandates required all state-licensed prescribers and dispensers to enroll in the relevant PMDP and registered prescribers to consult the PMDP for several clinical decisions [68]. Therefore, the utilization of mandates can offer an integral solution for addressing or reducing several public health challenges [67].

It is important to note that the overall goal of designing any intervention is being used in an organization’s routine operations, which becomes the measure of intervention success. Therefosre, designing for integration should be the goal for every mHealth developer. This implies that for the successful implementation continuum to be complete, an intervention must be used in the routine workflow of a health facility. This integration may be affected by factors such as resistance to use or interruption of the way things are performed in a health facility [69]. A study carried out in Thailand reported that the integration of a mobile application created additional tasks for healthcare providers [70]. It is important, therefore, that if the aspects of training, restructuring, incentivizing, and mandating are thoroughly effected, then integration in the routine workflows of a health facility becomes easy by addressing these issues.

### Strengths of the study

The TRIMI framework integrates domains and constructs from eight well-known implementation frameworks and implementation strategies to develop a framework that is specific to the sustainable implementation and integration of mHealth interventions.

### Limitations

This study is not without limitations; the framework has not yet been applied to practice, and there could be some hidden implementation and integration issues. However, we welcome additions and suggestions to this framework from researchers to enhance the effectiveness of the frameworks in terms of the sustainable implementation of mHealth innovations. The identified constructs may not be exhaustive.

It remains unclear which specific domain of the TRIMI is effective in facilitating the sustainable implementation and integration of mHealth interventions; therefore, effectiveness studies can be conducted by future researchers to concretize this framework. We believe that the TRIMI framework for mHealth intervention implementation will continue to evolve based on recommendations from implementers.

## Conclusion

We developed a framework that provides a well-developed approach for the sustainable implementation of mHealth interventions. We believe that the implementation of mHealth interventions generally depends on the purpose and the implementation environment, but the TRIMI framework can offer guidance for the sustainable implementation of mHealth interventions in low-resource settings. We call upon implementation scientists and researchers to explore the role of each specific construct as far as mHealth implementation is concerned to ascertain its effectiveness. We believe that the TRIMI framework for mHealth intervention implementation will continue to evolve based on recommendations from implementers, and more research can be done to ascertain the role of each individual domain in determining the effectiveness of mHealth intervention implementation.

## Figures and Tables

**Table 1:** Summary of the articles included in the scoping review for the final analysis (n = 8)

Name	Framework description	Domains	Definition of domains	Emergent TRIMI Domains
CFIR	Proposed by Damschroder and colleagues to guide systematic assessment, CFIR is a synthesis of 19 published implementation theories to develop a consolidated framework that provides a topology for promoting the implementation of interventions. CFIR has a record of accomplishment of providing consistent terminologies and the appropriate taxonomies for which implementation science can be built. CFIR has been updated and extended to 48 constructs and 19 sub constructs from the previous 39 constructs [34].	Intervention domain	A new strategy (education programme, new clinical treatment) being implemented	Integrate
		Outer setting	Involves external features (community, state, system) influencing the implementation of an intervention	
		Inner setting	Features (political, social, physical) of an organization in which the implementation process is effected	
		Individual’s domain	Characteristics and roles of individuals who influence the process of implementation	
		Implementation process domain	Involves tactics or set of activities that might facilitate intervention implementation.	
mCFIR	Is the mHealth version of the CFIR developed in 2015 by the University of British Columbia (UBC) mHealth Research Group [35]. The framework reframed the CFIR constructs from the mHealth perspective by adding a scoring system on each construct. It is made up of five key domains and two sub domains. The framework has the ability to identify, quantify and visualize the areas of strength and improvement.	Intervention characteristics	This involves the assessment of how well an intervention does or performs as intended	Training
		Outer settings	Involves the stakeholder’s environment in which implementation is occurring	
		Inner settings	Characteristics of an organization in which the implementation process is	
		End user characteristics	Opinions and perspectives of end users which include patients and healthcare workers	
		Implementation process	This involves evaluation and reflection or debriefing about the implementation before, during and after the implementation process. This is composed of two subdomains i) Goal attainment scale which assesses how well an organization is achieving its implementation goals and ii) the impact assessment scale that assesses how well an organizations achieving its key outcomes	
NPT	Concerned with the social organization of the work “implementation”, making practices routine elements of everyday life (embedding) and sustaining the embedded practices in their social context (integration) [71]. NPT is concerned with what individuals or groups of individuals do rather than what they believe or intend and has been utilized widely in healthcare settings [72]. The theory highlights four key components of implementation	Coherence	The practice that manipulates or organizes objects	Integrate
		Cognitive participation	Symbolic and real enrollments and engagement of human actors that positions them for the interactional and material work collective action	
		Collective action	Site of mental and material work that is about organizing and enacting a practice	
		Reflexive monitoring	Formal patterns of monitoring focus attention on normative elements of implementation	
UTAUT	UTAUT is the unification of eight models that help to explain the acceptance and use of technology [21]. These include Diffusion of Innovation Theory, Theory of Reasoned Action, Technology Acceptance Model, Theory of Planned Behavior, Combined Technology Acceptance Model, and Theory of Planned Behavior, Model of PC utilization and Social Cognitive Theory. UTAUT has been useful for assessing the likelihood of success for innovative technology and helps in understanding the factors for acceptance for proper designing of interventions. Understanding the feasibility and acceptability of the developed mHealth interventions is crucial in informing their adoption and usability.	Performance expectancy	The extent to which an individual believes that using a system will help him attain his job performance	Restructure
		Effort expectancy	The degree of ease associated with the use of the system	
		Social Influence	The extent to which an individual perceives how important others believe that they should use the new system	
		Facilitating conditions	The extent to which an individual believes that both the organizational and technical infrastructure exist to support the system’s use.	
COM-B	This model is a synthesis of 19 frameworks and contends that for a behavior to change, there is need for an interaction among capability, opportunity, and motivation of an individual. This guides in the understanding of the behavior thus leading to the design and development of the necessary behavioral targets that form a basis for the intervention design [73]. The model has a record of accomplishment in informing the design of interventions, for example Tuberculosis contact investigation [74] and tailoring interventions to address the context specific barriers [75].	Capability	Refers to the individuals’ psychological and physical ability (internal factors) to engage in a behavior	Train, Incentivize, Restructure
		Opportunity	Refers to an individual’s physical and social environment (external factors) which can facilitate or impede a behavior	
		Motivation	Involves all those automotive and reflective brain processes that energize and direct the behavior and is influenced by both capability and opportunity	
RE-AIM	Proposed by [24, 36], the framework categorizes five dimensions aimed at determining interventions that are worth investing in real-time environments and evaluating their effectiveness. The framework has been widely used in evaluating several public health interventions, for example eating disorders [76], and chronic illnesses [24]. It has been applied in understanding the use of mHealth technologies for promoting human papilloma vaccination [77, 78]	Reach	Proportion of the target population that participated in the intervention.	Restructure
		Efficacy	The rate of success if an intervention is implemented per the guidelines or the positive outcomes	
		Adoption	The proportion of settings, practices and plans that will be used to adopt an intervention.	
		Implementation	The extent to which an intervention is implemented in the real world	
		Maintenance	The extent to which a programme is sustained over time	
Theory of organizational readiness for change	Proposed by Weiner [37], this theory contends that the higher the organizational readiness for change, the more likely the organizational members are likely to initiate change, exert greater effort, exhibit greater persistence, and display more cooperative behavior, thus implementation effectiveness. The theory asserts that change is a collective behavior (commitment) and being able to change.	Change valence	The degree to which the members of the organization value the forthcoming change. As the members value the change, the more the desire to participate to implement the change	Restructure
		Change efficacy	The degree of judgment of perceived intervention’s capability to perform an individual task	
		Contextual factors	The degree to which an organization’s culture embraces intervention, risk-taking, and learning. For the culture within an organization can positively or negatively affect the implementation of the intervention.	
ERIC	Proposed by Powell and colleagues [38], provides a general experts’ consensus regarding the nomenclature for implementation strategies that can be used to implement intervention. It outlines 73 implementation strategies.	Access new funding, alter incentive/allowance structures, alter patient/consumer fees, assess for readiness and identify barriers and facilitators, audit and provide feedback, build a coalition, capture and share local knowledge, centralize technical assistance, change accreditation or membership requirements, change liability laws, change physical structure and equipment, change record systems, change service sites, conduct cyclical small tests of change, conduct educational meetings, conduct educational outreach visits, conduct local consensus discussions, conduct local needs assessment, conduct ongoing training, create a learning collaborative, create new clinical teams, create or change credentialing and/or licensure standards, develop a formal implementation blueprint, develop academic partnerships, develop an implementation glossary, develop and implement tools for quality monitoring, develop and organize quality monitoring systems, develop disincentives, develop educational materials, develop resource sharing agreements, develop resource sharing agreements, distribute educational materials, facilitate relay of clinical data to providers, facilitation, fund and contract for the clinical innovation, identify and prepare champions, identify early adopters, increase demand, inform local opinion leaders, intervene with patients/consumers to enhance uptake and adherence, involve executive boards, involve patients/consumers and family members, make billing easier, make training dynamic, mandate change, model and simulate change, obtain formal commitments, organize clinician implementation team meetings, place innovation on fee for service lists/formularies, prepare patients/consumers to be active participants, promote adaptability, promote network weaving, provide clinical supervision, provide local technical assistance, provide ongoing consultation, purposely reexamine the implementation, recruit, designate, and train for leadership, remind clinicians, revise professional roles, shadow other experts, stage implementation scale up, start a dissemination organization, tailor strategies, use advisory boards and workgroups, use an implementation advisor, use capitated payments, use data experts, use data warehousing techniques, use mass media, use other payment schemes, use train-the-trainer strategies, visit other sites, work with educational institutions		Incentivize, Train, Mandate

**Table 2:** Definitions of the domains and constructs of TRIMI

Domain	Definition	Constructs	Example
Train	Empowering users to use the intervention and educating them about its importance	• Intervention awareness	Workshops and seminars tailored to educate users about the importance of using the intervention.
		• Skills impartation	Group sessions with users by trained professionals to instill confidence in utilizing the intervention
Restructure	Improving or changing the physical or social context around an individual to influence their use of the intervention	• Technical and logistical support	Technical support and provision of logistics e.g., dedicated internet services and smartphones to users to ensure continued functionality of the intervention
		• Identifying committed staff	Acquiring new staff if necessary to act as change agents dedicated to support the intervention operationalization
		• Supervision	Supervision to ensure correct usage of the intervention by the development team
		• Redesigning	Management of recurring intervention design issues that may arise from the users e.g., fixing bugs, system upgrades
Incentivize	Motivating users to use the developed intervention	• Monetary incentives	Direct financial reimbursements to participants upon reaching a certain milestone
		• Nonmonetary incentives	Acknowledging the best users, and promotion of team-based performance
Mandate	Mandatory or compulsory authorization to use the intervention	• Organizational mandates	Internal policies, procedures within an organization and facility, aimed at ensuring that an intervention is used as intended
		• Government mandates	Compulsory government mandates regarding the use of the mHealth interventions
Integrate	Integration of the developed intervention into the clinical work routines within hospital settings and workflows within an organization	• Routine workflows	Embed the developed technology into the existing healthcare practice
		• Collaboration	Collaborative efforts from all relevant stakeholders for buy-in.

## Data Availability

The datasets used and/or analysed during the current study are available from the corresponding author on reasonable request
